# Exposure to airborne PM_2.5_ suppresses microRNA expression and deregulates target oncogenes that cause neoplastic transformation in NIH3T3 cells

**DOI:** 10.18632/oncotarget.5005

**Published:** 2015-08-21

**Authors:** Chunling Liu, Huan Guo, Xinxin Cheng, Mingming Shao, Chen Wu, Suhan Wang, Hongmin Li, Lixuan Wei, Yanning Gao, Wen Tan, Shujun Cheng, Tangchun Wu, Dianke Yu, Dongxin Lin

**Affiliations:** ^1^ State Key Laboratory of Molecular Oncology and Department of Etiology and Carcinogenesis, Cancer Institute and Hospital, Chinese Academy of Medical Science and Peking Union Medical College, Beijing, China; ^2^ Department of Occupational and Environmental Health and Ministry of Education Key Lab for Environment and Health, School of Public Health, Huazhong University of Sciences and Technology, Wuhan, China

**Keywords:** PM_2.5_, microRNA expression, gene expression, bronchial epithelial cell, neoplastic transformation

## Abstract

Long-term exposure to airborne PM_2.5_ is associated with increased lung cancer risk but the underlying mechanism remains unclear. We characterized global microRNA and mRNA expression in human bronchial epithelial cells exposed to PM_2.5_ organic extract and integrally analyzed microRNA-mRNA interactions. Foci formation and xenograft tumorigenesis in mice with NIH3T3 cells expressing genes targeted by microRNAs were performed to explore the oncogenic potential of these genes. We also detected plasma levels of candidate microRNAs in subjects exposed to different levels of air PM_2.5_ and examined the aberrant expression of genes targeted by these microRNAs in human lung cancer. Under our experimental conditions, treatment of cells with PM_2.5_ extract resulted in downregulation of 138 microRNAs and aberrant expression of 13 mRNAs (11 upregulation and 2 downregulation). In silico and biochemical analyses suggested *SLC30A1*, *SERPINB2* and *AKR1C1*, among the upregulated genes, as target for miR-182 and miR-185, respectively. Ectopic expression of each of these genes significantly enhanced foci formation in NIH3T3 cells. Following subcutaneous injection of these cells into nude mice, fibrosarcoma were formed from *SLC30A1*- or *SERPINB2*-expressing cells. Reduced plasma levels of miR-182 were detected in subjects exposed to high level of PM_2.5_ than in those exposed to low level of PM_2.5_ (*P* = 0.043). Similar results were seen for miR-185 although the difference was not statistically significant (*P* = 0.328). Increased expressions of *SLC30A1*, *SERPINB2* and *AKR1C1* were detected in human lung cancer. These results suggest that modulation of miR-182 and miR-185 and their target genes may contribute to lung carcinogenesis attributable to PM_2.5_ exposure.

## INTRODUCTION

Epidemiologic studies have associated increased lung cancer risk with long-term exposure to airborne particulate matters (PMs) including PM_2.5_ [1, 2], which contains mixture of crustal elements, metals, elemental carbon, inorganic ions (e.g., sulfate, nitrate, and ammonium), organic compounds (e.g., polycyclic aromatic hydrocarbons), and biogenic species [2–5]. It has been shown that PM_2.5_ can induce genetic and epigenetic changes in the airway tissues [6–10]. Some previous studies have characterized the effects of PM_2.5_ on the human airway epithelial transcriptome and found that PM_2.5_ may induce the expression of airway genes involved in oxidative stress, xenobiotic metabolism and oncogenesis, but suppress the expression of genes involved in immune response and tumor suppression [11–13]. These findings indicate that the changes in gene expression in airway tissues may reflect host response to and damage from PM_2.5_. However, the mechanism underlying these gene expression changes and their consequences remains to be further elucidated.

MicroRNAs are a large family of post-transcriptional regulators of gene expression and they have been predicted to control the activity of about 50% of all protein-coding genes in mammals. It has been suggested that microRNAs participate in the regulation of almost every cellular process and the change in their expression are associated with many human diseases [14]. MicroRNA expression has been shown to be altered by exposure to air pollutants including PM_2.5_. One previous study reported an association between exposure to PM_2.5_ and downregulation of several select microRNAs in elderly men [15]. Another study showed that single nucleotide polymorphisms in microRNA-processing genes modify the association between ambient pollutants including PM_2.5_ and soluble intercellular adhesion molecule-1 and vascular cell adhesion molecule-1 levels, which are associated with atherosclerosis and cardiovascular disease [16]. These findings imply that altered microRNA expression may play a role in PM_2.5_-induced diseases. However, up to date, there is little experimental evidence showing the effects of exposure to airborne PM_2.5_ on microRNA expression in cells and its biological consequences.

In this study, we hypothesized that components of airborne PM_2.5_ may alter microRNA expression resulting in deregulation of their target oncogenes, which in turn may lead to carcinogenesis. To test this hypothesis, we first examined the effects of PM_2.5_ on whole-genome microRNA expression in human bronchial epithelial (HBE) cells. We then identified protein-coding genes that may be targeted by PM_2.5_-modulated microRNAs. Furthermore, we investigated the phenotypic changes in mouse NIH3T3 cells ectopically and stably expressing these microRNA-targeted genes. We also detected the plasma levels of the candidate microRNAs in subjects exposed to different levels of ambient airborne PM_2.5_, and examined the expression of these microRNA-targeted genes in human lung cancer. Here we show that exposure to PM_2.5_ suppressed some microRNA expression *in vitro* and *in vivo*. The suppression of these microRNAs elevated expression of their target genes that may cause neoplastic transformation in NIH3T3 cells. Low plasma levels of candidate microRNAs and aberrant expression of their target genes were detected in human subjects exposed to higher levels of airborne PM_2.5_ and lung cancer tissues, respectively.

## RESULTS

### Alterations of microRNA and mRNA expression in HBE cells exposed to PM_2.5_ extract

**Figure 1:**
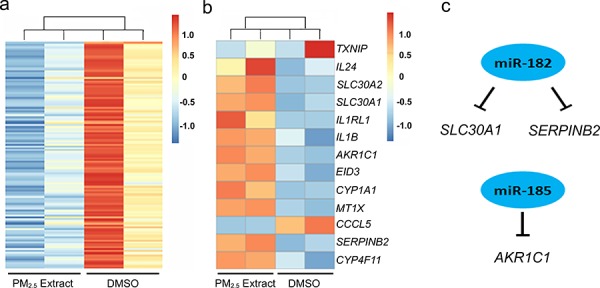
Altered global microRNA a. and mRNA b. expression in human bronchial epithelial cells exposed to DMSO extracts of airborne PM_2.5_, and potential interactions between microRNAs and mRNAs suggested by integrate and in silico analysis c. The experimental conditions are described in Materials and Methods

Under our experimental conditions and analytical criteria as described in Method, there were 13 genes aberrantly expressed (11 upregulated and 2 downregulated) in HBE cells exposed to dimethylsulfoxide (DMSO) extracts of PM_2.5_ compared with those in cells without exposure (Figure 1a). These genes are functionally involved in xenobiotic metabolism (e.g., *CYP1A1*, *CYP4F11*, *AKR1C1*, *MT1X*, *TXNIP*, *SLC30A1* and *SLC30A2*), immunoregulatory or inflammatory response (e.g., *IL24*, *IL1RL1*, *IL1B*, *CCL5 and SERPINB2*) and other (*EID3*). Meanwhile, we identified 138 microRNAs aberrantly expressed in exposed cells compared with unexposed cells and all of these microRNAs were downregulated (Figure 1b). We then examined the correlation between the expression of microRNAs and mRNAs to predict the potential targets of microRNAs and found 3 genes, *SLC30A1*, *SERPINB2* and *AKR1C1*, whose expression was inversely correlated with miR-182 and (or) miR-185 expression (Figure 1c). These results suggested that *SLC30A1*, *SERPINB2* and *AKR1C1* may be regulatory targets for miR-182 and miR-185. The upregulation of *SLC30A1*, *SERPINB2* and *AKR1C1* and downregulation of miR-182 and miR-185 in HBE cells exposed to PM_2.5_ extract detected by microarray were further confirmed by using quantitative real-time PCR (qRT-PCR) and Western blot assays (Supplementary Figure S1a and S1b).

### *SLC30A1*, *SERPINB2* and *AKR1C1* are *bona fide* target genes of miR-182 or miR-185

**Figure 2:**
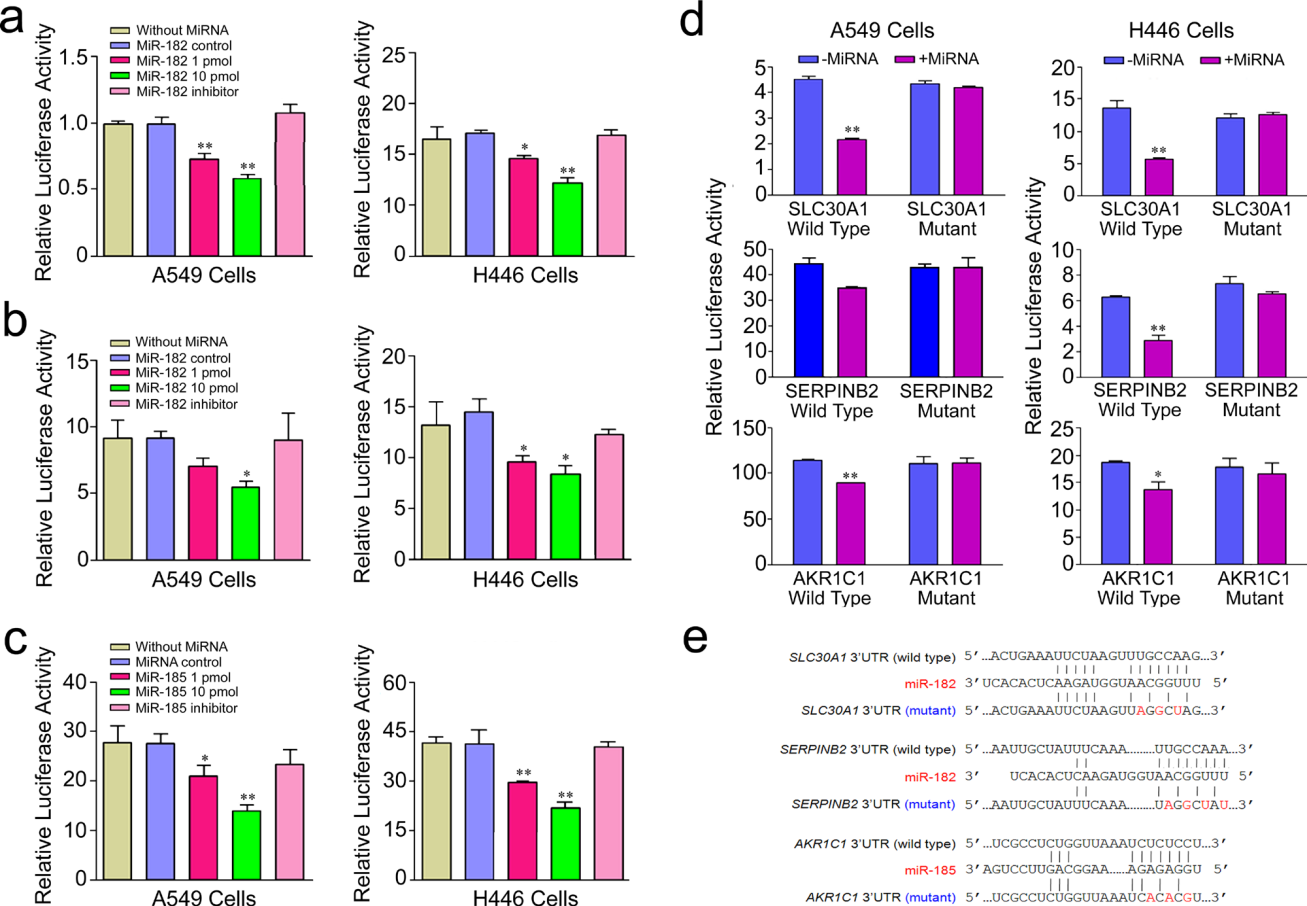
Relative activity of reporter gene constructed with wild type of 3′UTR of *SLC30A1* a. *SERPINB2* b. or *AKR1C1* c. gene or their mutant types d. cotransfected with miR-182 or mir-185 or their inhibitors in A549 and H446 cells. Results are mean ± SEM obtained from three experiments and each had six replicates. *, *P* < 0.05 and **, *P* < 0.01 compared with without microRNA control or wild type. Mutations in the core microRNA binding sites are shown e

**Figure 3:**
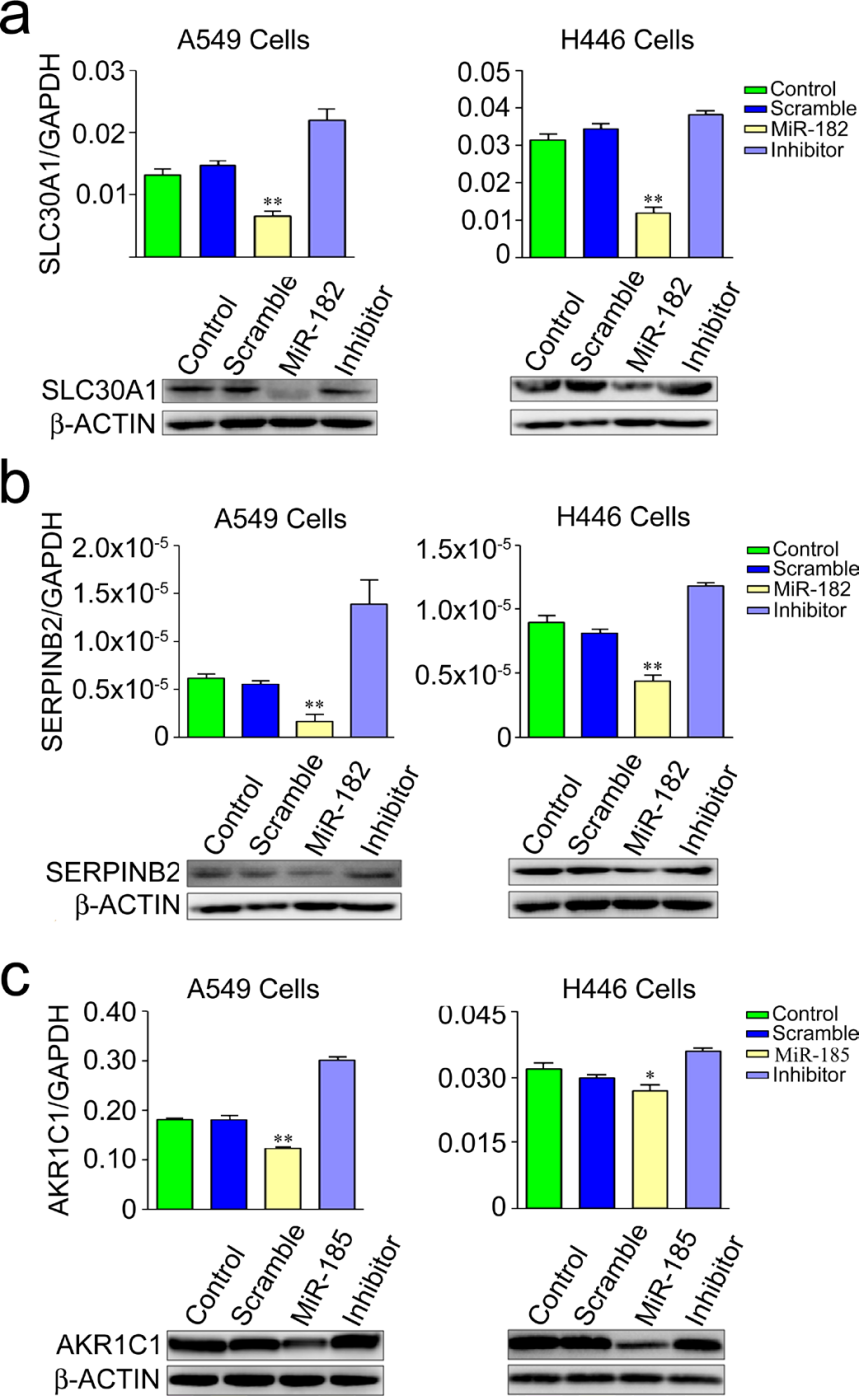
Suppression of endogenous mRNA (*up panel*) and protein (*lower panel*) of *SLC30A1* a. *SERPINB2* b. and *AKR1C1* c. in A549 and H446 cells transfected with miR-182 mimic, miR-185 mimic or their inhibitor. Results of mRNA levels are mean ± SEM obtained from three experiments. *, *P* < 0.01 and **, *P* < 0.001 compared with control or inhibitor

To test whether *SLC30A1*, *SERPINB2* and *AKR1C1* are *bona fide* targets of miR-182 or miR-185, a series of assays were conducted. First, we constructed luciferase reporter plasmids with 3′UTR of the *SLC30A1*, *SERPINB2* and *AKR1C1* genes, respectively, in psiCHECK-2 vector. Transient transfection of these reporter plasmids to human lung cancer cell lines A549 and H446 with miR-182 or miR-185 mimic or microRNA control showed that transfection with miR-182 significantly reduced the luciferase activity caused by 3′UTR of *SLC30A1* or *SERPINB2* while transfection with miR-185 significantly reduced luciferase activity caused by 3′UTR of *AKR1C1* (all *P* < 0.05). The reduction of luciferase activity was in a microRNA concentration-dependent manner in both A549 and H446 cells and when the microRNA inhibitor was presented, the reduction was completely rescued (Figure 2a, 2b and 2c). We next constructed luciferase reporter plasmids with 3′UTR of *SLC30A1*, *SERPINB2* or *AKR1C1* mutated in the core microRNA binding sites by site-directed mutagenesis (Figure 2e). Transfection of these plasmids with miR-182 or miR-185 mimic showed no significant change in luciferase activity compared with transfection of these plasmids with microRNA control (Figure 2d), suggesting that the interactions between the two microRNAs and 3′UTR of three target genes are sequence-specific. Furthermore, the significant suppression of both endogenous mRNA and protein expression of *SLC30A1*, *SERPINB2* or *AKR1C1* in A549 and H446 cells was verified by transfection of cells with miR-182 or miR-185 mimic (all *P* < 0.01), and this microRNA-induced suppression of gene expression could be rescued when the specific miRNA inhibitor was co-transfected (Figure 3a–3c). These results provided further evidence that *SLC30A1*, *SERPINB2* and *AKR1C1* are respective *bona fide* target genes of miR-182 or miR-185 in human cells.

### Overexpression of SLC30A1 or SERPINB2 evokes neoplastic transforming in NIH3T3 cells

**Figure 4:**
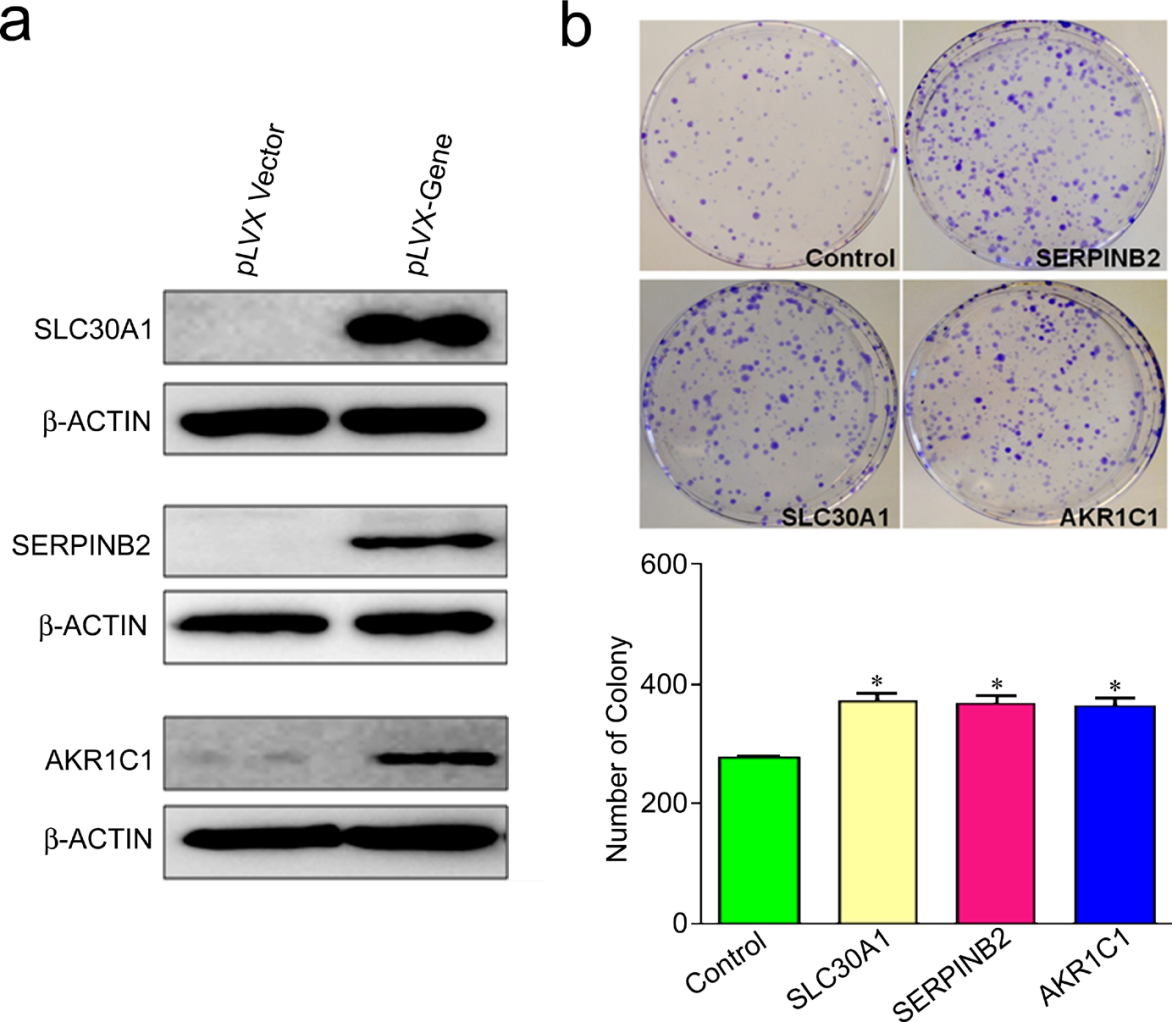
Foci formation ability of SLC30A1, SERPINB2 or AKR1C1 ectopically and stably expressed in NIH3T3 cells. a. Establishment of NIH3T3 cells stably expressing SLC30A1, SERPINB2 or AKR1C1. b. Colony number (mean ± SE) of cells with ectopic expression of each of the three genes. *, *P* < 0.05 compared with vector control

**Figure 5:**
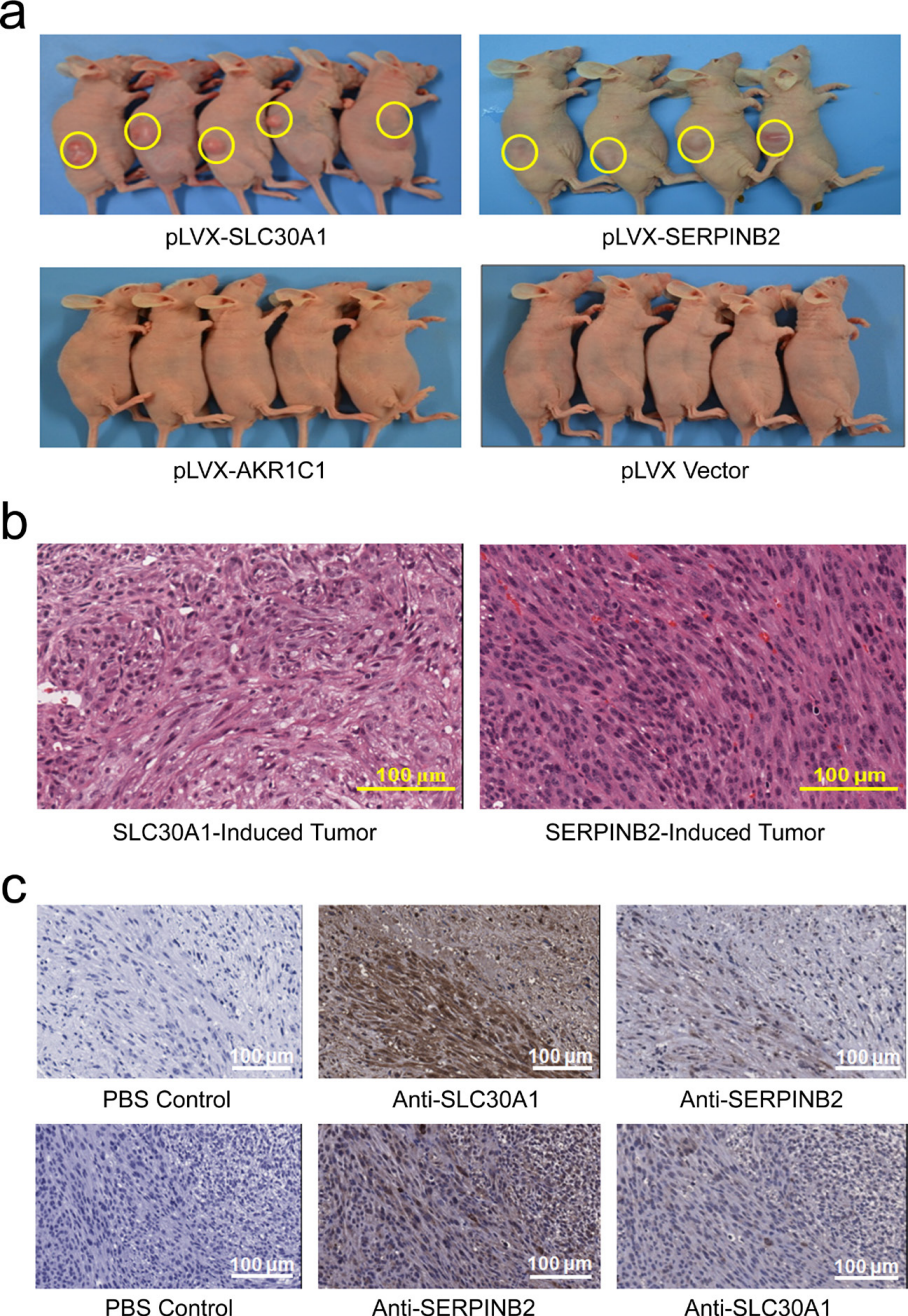
Xenograft tumor formation in nude mice of NIH3T3 cells ectopically and stably expressing SLC30A1, SERPINB2, AKR1C1, or vector control a. Histological analysis showed that all tumor cells had similar morphology and were diagnosed as fibrosarcoma b. Immunohistochemical staining c. demonstrated a high expression of SLC30A1 (*up panel*) or SERPINB2 (*lower panel*), respectively, in tumors induced by each of these two genes

Mouse NIH3T3 cells ectopically and stably expressing SLC30A1, SERPINB2 or AKR1C1 were established (Figure 4a) to test the neoplastic transforming activity of these genes. We first conducted foci formation assay and found that colony number (mean ± SE) of cells with ectopic expression of each of SLC30A1 (389.3 ± 10.7, *P* = 0.002), SERPINB2 (370 ± 9.1, *P* = 0.003) or AKR1C1 (354 ± 7.9, *P* = 0.006) was significantly greater than that of cells with vector control (289.7 ± 9.1) (Figure 4b). We then examined tumorigenesis of these NIH3T3 cells *in vivo* by subcutaneous injection to nude mice. Although no tumor was found in animals (*n* = 5) injected with cells transfected with vector, all animals injected with cells expressing SLC30A1 (*n* = 5) or SERPINB2 (*n* = 4) developed tumor at the xenograft site within 4 weeks after injection. However, under the same experimental conditions, injection of cells expressing AKR1C1 to mice (*n* = 5) failed to induce tumor (Figure 5a). Histological analysis showed that all the tumor cells had similar morphology and were diagnosed as fibrosarcoma (Figure 5b). Immunohistochemical staining confirmed that SLC30A1 and SERPINB2 were highly expressed in tumors induced by NIH3T3 cells transfected with human *SLC30A1* or *SERPINB2*, respectively (Figure 5c).

### Reduced expression of miR-182 and miR-185 in human subjects exposed to PM_2.5_

Plasma miR-182 and miR-185 were measured in 109 subjects living at the same region (Wuhan, China) but exposed to different levels of PM_2.5_ and PM_10_ monitored by personal sampler for 24 h. The median levels of individuals' exposure to PM_2.5_ and PM_10_ were 124.8 μg/m^3^ and 179.3 μg/m^3^, respectively, with the ranges of 18.7 to 274.2 μg/m^3^ for PM_2.5_ and 39.8 to 383.3 μg/m^3^ for PM_10_ (Supplementary Table S1). We then separately stratified all subjects into low exposure (≤124.8 μg/m^3^ PM_2.5_ or ≤179.3 μg/m^3^ PM_10_) and high exposure (>124.8 μg/m^3^ PM_2.5_ or > 179.3 μg/m^3^ PM_10_) groups based on the median levels of PM_2.5_ or PM_10_ exposure. The results showed that median plasma levels of miR-182 were significantly decreased in subjects exposed to high levels of PM_2.5_ and PM_10_ compared with that in subjects exposed to low levels of PM_2.5_ (0.166 versus 0.411, *P* = 0.043) and PM_10_ (0.141 versus 0.457, *P* = 0.007), respectively. For miR-185, the reduced levels in subjects with high exposure compared with those with low exposure were also observed although the differences were not statistically significant (Table 1).

**Table 1:** Plasma miR-182 and miR-185 in subjects exposed to different levels of PM_2.5_ and PM_10_

MicroRNA	PM_2.5_ (μg/m^3^)		PM_10_ (μg/m^3^)	
	≤124.8 (*N* = 62)	>124.8 (*N* = 47)	≤179.3 (*N* = 60)	>179.3 (*N* = 49)
**MiR-182 (× 10^−4^)^a^**				
Median (25%, 75%)	0.411 (0.097, 0.887)	0.166 (0.062, 0.590)	0.457 (0.108, 0.990)	0.141 (0.062, 0.519)
β (95% CI)	Reference	−0.905 (−1.780, 0.030)	Reference	−1.191 (−2.063, 0.319)
*P*-Value^b^	Reference	0.043	Reference	0.007
**MiR-185 (× 10^−3^)^a^**				
Median (25%, 75%)	0.573 (0.216, 1.442)	0.468 (0.151, 1.450)	0.573 (0.205, 1.772)	0.468 (0.170, 1.367)
β (95% CI)	Reference	−0.454 (−1.363, 0.455)	Reference	−0.339 (−1.259, 0.581)
*P*-Value^b^	Reference	0.328	Reference	0.470

a Relative expression normalized to cel-miR-39 and calculated by 2^−ΔCt^.

b Multivariate regression analysis with adjustment for age, sex, smoking status, drinking status, and body mass index.

### Aberrant expression of *SLC30A1*, *SERPINB2* and *AKR1C1* in human lung cancer

**Figure 6:**
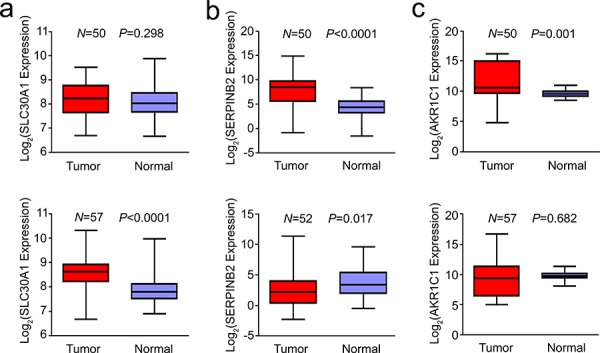
Aberrant expression of *SLC30A1* a. *SERPINB2* b. and *AKR1C1* c. in human lung cancer and paired normal tissues (*up panel*, squamous cell carcinoma and *lower panel*, adenocarcinoma) Data were retrieved from the TCGA database

We further analyzed *SLC30A1*, *SERPINB2* and *AKR1C1* mRNA expressions in human lung cancer retrieved from the TCGA database. It was found that the median *SLC30A1* RNA level in lung tumor tissues was significantly higher than that in normal tissues (8.459 versus 7.837, *P* < 0.0001); however, this significant difference was only occurred in adenocarcinoma but not in squamous cell carcinoma (Figure 6a). The median *SERPINB2* RNA level in lung tumor tissues was also significantly higher than that in normal tissues (4.625 versus 3.950, *P* = 0.0053) and this difference was predominant in squamous cell carcinoma (Figure 6b). For *AKR1C1* RNA, higher expression levels in tumor tissues were seen in squamous cell carcinoma (10.530 versus 9.525, *P* = 0.0013) but not adenocarcinoma (Figure 6c).

## DISCUSSION

In the present study, we showed that down-regulation of miR-182 and miR-185 in HBE cells exposed to DMSO extracts of PM_2.5_ resulted in increased *SLC30A1*, *SERPINB2* and *AKR1C1* gene expression and ectopic expression of these genes can respectively lead to neoplastic transformation in NIH3T3 cells. We also detected depressed expression of miR-182 and perhaps miR-185 in human subjects exposed to high level of PM_2.5_ and overexpression of *SLC30A1*, *SERPINB2* and *AKR1C1*, which we have first demonstrated to be target genes of miR-182 and/or miR-185, in human lung cancer compared with the corresponding normal lung tissues. These results together suggest that exposure to airborne PM_2.5_ may cause depression of some microRNAs, which results in overexpression of their target oncogenes and consequently leads to lung carcinogenesis. To the best of our knowledge, this is the first report comprehensively examining the effects of PM_2.5_ organic extract on whole-genome expressions of microRNAs and protein-coding genes and their association with lung cancer.

Several previous studies have examined PM_2.5_ and PM_10_ on global gene expression profile *in vitro* in cell cultures. Despite differences in cell types, microarray platforms, and PM sources and doses, these experiments showed commonality in the expression of genes and pathways such as xenobiotic metabolism, immune or inflammation, and oxidative stress [11–13]. In the present study, we identified 13 protein-coding genes that had 2-fold change in their expression in HBE cells treated with PM_2.5_ DMSO extract compared with control (11 up-regulated and 2 down-regulated). These genes also are mainly involved in xenobiotic metabolism and immunoregulatory or inflammatory pathways and some of them have been reported in the published studies [11–13]. In addition to protein-coding genes, we found aberrant expression of 138 microRNAs in HBE cells exposed to PM_2.5_ DMSO extract under the same experimental conditions, and all of them were downregulated. Although the underlying mechanism has yet to be clarified, it is reasonable to speculate that downregulation of microRNA expression might be necessary for cells to activate some key genes involved in xenobiotic metabolism, DNA repair, immune, inflammation and proliferation pathways to maintain cell survival upon exposure to toxic environmental chemicals. Similar phenomenon has been observed in previous studies showing that the expression of most microRNAs were declined *in vivo* in human subjects exposed to ambient PMs [15] or cigarette smoke [17] or *in vitro* in cells treated with certain chemical [18].

The most significant finding in the present study is that we identified at the first time that miR-182 and miR-185 are target regulators of *SLC30A1*, *SERPINB2* or *AKR1C1* that function as potential oncogenes because they were able to induce neoplastic transformation in NIH3T3 cells and were overexpressed in human lung cancer. We also observed reduced levels of plasma miR-182 and perhaps miR-185 in human subjects exposed to high levels of PM_2.5_ compared with those exposed to low levels of PM_2.5_ residing at the same region, directly connecting PM_2.5_ exposure to microRNA expression *in vivo*. It has been shown that miR-182 is overexpressed in many types of cancer including lung cancer and is thought to be associated with cancer development and prognosis [19, 20]. However, little has been known about the effect of miR-182 on lung cancer initiation. Accumulating evidence has shown that microRNAs have distinct effects in different biological contexts. Indeed, miR-182 has also been shown to act as a tumor suppressor. For example, miR-182 was reported to suppress lung tumorigenesis and lung cancer cell proliferation through downregulation of *RGS17* or *RASA1* [21, 22]. In another study, downregulation of miR-182 was shown to contribute to renal cell carcinoma proliferation via activation of AKT/FOXO3a signaling pathway [23]. MiR-185 seems to be a tumor suppressor and is frequently downregulated in many types of human cancer [24–26]. Taken together, these results suggest that long-term exposure to PM_2.5_ may suppress some microRNA expression, which evokes the activation of certain oncogenes and thus results in developing malignancies such as lung cancer.

In the present study, we identified *SLC30A1*, *SERPINB2* and *AKR1C1* as potential oncogenes targeted by miR-182 and (or) miR-185, respectively. We demonstrated that ectopic and stable expression of these genes in NIH3T3 cells could induce cell neoplastic transformation. Intracellular zinc homeostasis, which plays an important role in maintaining normality of cells, is tightly controlled by two families of zinc transporters, SLC39 and SLC30. It has been shown that cytosolic zinc negatively regulates RAS-mediated signaling and physiological zinc level is important for maintaining the inactive state of the RAS pathway [27]. On the other hand, it was reported that exposure to zinc sulfate in human prostate cancer cells increased intracellular levels of zinc, resulting in increased apoptosis, which could be due to increased levels of BAX or decreased Bcl-2 and survivin expression [28]. SLC30A1 is a SLC30 family member acting as primary regulator of cellular zinc efflux and overexpression of SLC30A1 may disrupts intracellular zinc homeostasis, leading to cell neoplastic transformation. *SERPINB2*, encoding plasminogen activator inhibitor type 2 (also known as PAI-2), is commonly expressed in many types of human cancer including lung cancer [29]. The expression of *SERPINB2* in human cells can be induced by chemical carcinogens such as cigarette smoke and dioxin [30–32]. It has been shown that overexpression of *SERPINB2* in keratinocytes enhances papilloma formation in transgenic mice [33] and knockout of *SERPINB2* abrogates papilloma formation in mice [34]. The findings in these previous studies support our results that *SERPINB2* can be induced by PM_2.5_ and ectopic expression of this gene is implicated in neoplastic transformation in NIH3T3 cells. *AKR1C1* gene encodes a member of the aldo/keto reductase superfamily, which catalyze the conversion of aldehydes and ketones to generate their corresponding alcohols. Previous studies showed that in human lung cell lines, AKR1C1 and AKR1A1 also play an important role in the metabolic activation of polycyclic aromatic hydrocarbons, a major component in PM_2.5_, which may contribute to the causation of human lung cancer [35]. In fact, it has been documented that AKR1C family including AKR1C1 are able to drive neoplastic transformation of NIH3T3 cells [36], which is generally consistent with our results in the present study, although we failed to induce tumor in nude mice with transformed NIH3T3 cells having significantly increased colony-forming ability.

The analysis of *SLC30A1*, *SERPINB2* and *AKR1C1* levels in human lung specimens from the TCGA database also revealed the aberrant expression of these genes in lung cancer tissues compared with paired normal tissues. Although these results did not provide direct evidence that the aberrant expression of *SLC30A1*, *SERPINB2* and *AKR1C1* in human lung cancer is caused by exposure to PM_2.5_, they did indicate a role in the development of lung cancer of these genes whose expression can be induced by PM_2.5_ organic extract. Interestingly, we found that the aberrant expression of these genes differed in lung adenocarcinoma and squamous cell carcinoma, suggesting that they may function differentially in carcinogenesis of different lung cancer subtypes. Further studies are needed to address this issue.

The present study had some limitations. First, because we used DMSO as solvent to extract PM_2.5_, therefore the results reflected only the effects of components dissolved in DMSO. Since PM_2.5_ may contain numerous carcinogens that have different chemical/physical features depending on the sources of air pollutants, it would be interesting to investigate the effects of other components in PM_2.5_ using other extracts. Second, although the ectopic expression of *SLC30A1*, *SERPINB2* and *AKR1C1* can drive neoplastic transformation of NIH3T3 cells, whether they can drive neoplastic transformation of human cells and what are the underlying mechanisms need to be examined. Furthermore, in this study, we identified aberrant expression of 138 microRNAs and 13 protein-coding genes and examined only 2 microRNAs and their 3 target genes; it would be interesting to investigate the oncogenic potential of other miRNAs in the future. Lastly, the sample sizes for investigating the associations between PM_2.5_ exposure and plasma miRNAs were relatively small, thus the findings need further validation with larger sample sizes.

In summary, we have identified a bunch of microRNAs and protein-coding genes that were aberrantly expressed in human bronchial epithelial cells exposed to DMSO extracts of airborne PM_2.5_. Among them, overexpression of *SLC30A1*, *SERPINB2* as well as *AKR1C1*, mediated by downregulation of miR-182 and (or) miR-185, can induce neoplastic transformation in NIH3T3 cells. Depression of plasma miR-182 and miR-185 in subjects exposed to high levels of PM_2.5_ and overexpression of *SLC30A1*, *SERPINB2* and *AKR1C1* in human lung cancer tissues were detected. These results suggest that altered expression of microRNAs and their target oncogenes may contribute to the development of lung cancer attributable to airborne PM_2.5_.

## MATERIALS AND METHODS

### Study subjects and individual monitoring of airborne PM exposure

A pilot study of 109 subjects with the availability of personal 24-h exposure to ambient PM_2.5_ and PM_10_ and blood were recruited from a community-based, prospective cohort study conducted between April and May of 2011 in Wuhan City, China [37]. These subjects resided in two distinct communities of Wuhan City; one is located in the urban district and the other is in the suburb. All subjects have been living in the sampling buildings for >5 years and aged >40 years, free of chronic diseases based on physical examination [37]. Each individual was interviewed by using a structure questionnaire including tobacco smoking, alcohol use, and environment exposure history. Twenty four-hour exposure of subjects to PM_2.5_ and PM_10_ was monitored by using a Model 200 Personal Environmental Monitor (MSP, Minnesota) and Gilian 5000 pump (Sensidyne, Florida). PM samples were collected on 37-mm Teflon filters (LianyiXingtong Apparatus & Instrument, Beijing) at the flow rate of 2.0 l/min. Before and after sampling, the filters were weighted after conditioning for 24 h. A 24-h PM_2.5_ or PM_10_ exposure level was calculated to represent as personal exposure level. At recruitment, informed consent for the study, use of blood sample and medical records was obtained from each subject. This study was approved by the Institutional Review Board of the Huazhong University of Sciences and Technologies School of Public Health.

### Preparation of PM_2.5_ extract

Urban atmospheric PM_2.5_ was collected in Wuhan City between April and May of 2011 by using a PM_2.5_ sampler as described above. PM_2.5_ extract was prepared by suspending PM_2.5_ sample in dimethylsulfoxide (DMSO) at a proportion of 200 mg/ml. The mixture was extracted in an ultrasonic bath for 30 min and then briefly centrifuged to obtain the supernatant, which was stored at −40°C until use.

### Cell culture and treatment

Immortalized human bronchial epithelial (HBE) cells established in our laboratory [38] were maintained in serum-free medium. Mouse fibroblast cells (NIH3T3), human lung adenocarcinoma (A549) cells and small-cell lung cancer (H446) cells, purchased from the Cell Bank of Type Culture Collection of Chinese Academy of Sciences Shanghai Institute of Biochemistry and Cell Biology (Shanghai, China), were maintained according to the Cell Bank's protocols. Cell lines were characterized by DNA finger printing analysis using short-tandem repeat markers. HBE cells (1.5 × 10^5^) were seeded in six-well plates and allowed to grow for 24 h in an incubator at 37°C with 4% CO_2_. PM_2.5_ extract was added to the cell culture at a final concentration of 0.45 mg/ml, a dose that allows 90% of HBE cells alive after exposure for 24 h. Cells treated with equal amount of DMSO served as solvent control.

### Isolation of RNA from plasma, cells and lung tissues

For plasma, total RNA was isolated from 200 μl of EDTA-anticoagulated sample according to the manufacturer's protocol of the mirVana PARIS miRNA Isolation Kit (Ambion 1556, Austin, TX). Twenty five fmol of Caenorhabditis elegans cel-miR-39 (synthesized by Qiagen) was added to each sample as the internal control. For HBE cells and lung tissue samples, total RNA was extracted with Trizol (Invitrogen, Carlsbad, CA) according to the manufacturer's instructions. RNA integrity was assessed using a 2100 Bioanalyzer (Agilent, Santa Clara, CA).

### Microarray analysis of mRNA and microRNA expression

Glue Grant Human Transcriptome Array (Affymetrix, Santa Clara, CA) and Human miRNA Microarray 8 × 60 K platform version 18 (Agilent) were used to generate whole-genome expression profiles of mRNAs and microRNAs, respectively. We set a threshold of 2-fold change (exposed versus control) to obtain mRNA profile and 1.5-fold change to obtain microRNA profile as significantly induced or repressed by PM_2.5_ extract. The experiments were performed in duplicate. The integrated mRNA and microRNA expression data were used to identify the potential microRNA target genes by using the Ingenuity Pathway Analysis (IPA) software as well as TargetScan and miRanda databases.

### Quantitative real-time PCR analysis of candidate mRNAs and microRNAs

First-strand cDNA was synthesized with the Superscript II-reverse transcriptase kit (Invitrogen) using total RNA as the template. For analysis of microRNAs from cell lines and tissue samples, polyadenylation was performed before cDNA synthesis. Quantitative real-time PCR (qRT-PCR) was accomplished on an ABI Prism 7900 sequence detection system (Applied Biosystems, Foster City, CA) based on the SYBR Green method and *GAPDH* as the internal control. The expression of miR-182 and miR-185 was calculated relative to U6 small nuclear RNA. For analysis of plasma miR-182 and miR-185, the input RNA was reverse transcribed using TaqMan miRNA Reverse Transcription Kit (Applied BioSystems, Foster City, CA). The miRNA levels were normalized to cel-miR-39. The gene- and microRNA-specific qRT-PCR primer pairs are shown in Supplementary Table S2.

### Reporter plasmid construction, transient transfection and luciferase assay

The 3′ untranslated region (3′UTR) of *SLC30A1*, *SERPINB2*, which were generated by PCR, or *AKR1C1*, which was synthesized (Genewiz, South Plainfield, NJ), was subcloned in psiCHECK2 vector (Promega, Fitchburg, WI) as reporter plasmids. Mutants of these 3′UTR fragments at the microRNA binding seed site were achieved by using the Site-Directed Mutagenesis kit (SBS Genetech, Beijing). All constructs were restriction-mapped and sequenced to confirm their authenticity. A549 and H446 cells were seeded at 1 × 10^5^ cells per well in 24-multiwell plates (BD Biosciences, San Jose, CA) and allowed to grow to 80%–90% confluence. Reporter plasmids (100 ng) with microRNA mimics or scrambled sequences of microRNA inhibitors were co-transfected to cells using lipofectamine 2000 (Invitrogen). Each group had three replicates, and the experiment was repeated at least three times. Cells were collected at 24 h after transfection using 100 μl of passive buffer and Renilla luciferase activity was detected with Dual-Luciferase Reporter Assay System (Promega) in a TD-20/20 luminometer (Turner Biosystems, Sunnyvale, CA); firefly luciferase activity was also determined to normalize the activity of Renilla luciferase.

### Expression plasmid construction, lentiviral production and transduction

The expression plasmids pLVX-SLC30A1, pLVX-SERPINB2 and pLVX-AKR1C1 were constructed using pLVX-IRES-Neo lentiviral vector and full length cDNAs of human *SLC30A1*, *SERPINB2* generated by PCR (see Supplementary Table S2 for the primers), or *AKR1C1* provided by OriGene Technologies (Rockville, MD). These expression plasmids were used to generate recombinant lentiviruses in HEK293T cells by cotransfection with VSVG, REV and ΔR. Transfection of pLVX-SLC30A1, pLVX-SERPINB2, pLVX-AKR1C1 or empty pLVX vector into NIH3T3 cells was done using lipofectamine 2000 DNA Transfection Reagent (Invitrogen). At 48 h of transfection, cells were stably selected with G418 and drug-resistant cells were used for subsequent studies.

### Western blotting assay

Western blotting assay was used to detect the protein expression levels of interest genes in HBE cells exposed to PM_2.5_ extract or cells transfected with expression plasmids containing the interest genes. Protein extract prepared by detergent lysis was subjected to SDS-polyacrylamide gel electrophoresis and transferred to PVDF membrane (Millipore, Temecula, CA). Antibodies against SLC30A1 (ab110383, Abcam, Cambridge, MA), SERPINB2 (16035-1-AP, ProteinTech, Chicago, IL), AKR1C1 (7660-1, Epitomics, Burlingame, CA) or β-ACTIN (20536-1-AP, ProteinTech) were used. The membranes were incubated overnight at 4°C with the primary antibody and the proteins were then detected with a Phototope-horseradish peroxidase Western blot detection kit (Cell Signaling Technology).

### Colony formation assay

NIH3T3 cells with ectopic and stable expression of human SLC30A1, SERPINB2 or AKR1C1 were plated evenly onto 10-cm dishes in medium containing 10% FBS. Cells were fed every three days over 10 days in culture. Colonies were fixed in cold methanol and stained with crystal violet.

### Xenograft growth of NIH3T3 cells in mice

BALB/c nude mice, aged 6 weeks and purchased from Shanghai Laboratory Animal Center of the Chinese Academy of Sciences, were allowed to acclimatize to local conditions for one week and maintained under a 12-h dark, 12-h light cycle with food and water ad libitum. Animals (5 mice in each group) were injected subcutaneously with 0.1 ml of cell suspension containing 10^7^ cells into the right flank. When a tumor was palpable, its volume was measured every other day and calculated according to the formula, Volume = Length × Width^2^ × 0.5. All experiments were performed in accordance with the Institutional Review Board of the Cancer Institute and Hospital and national guidelines and regulations.

### Histological and immunohistochemical analysis

Formalin-fixed, paraffin-embedded tumor samples were sectioned, stained with hematoxylin and eosin and analyzed under light microscopy. To investigate the presence of SLC30A1 or SERPINB2 proteins in tumor tissues, the rabbit antibody against SERPINB2 or SLC30A1 (1:500 dilution) was incubated with the tissue sections at 4°C overnight, which was then detected with the ABC kit (Pierce).

### Retrieval of data from online databases

RNA expression levels of *SLC30A1*, *SERPINB2* and *AKR1C1* in lung cancer and their paired normal tissues were obtained from The Cancer Genome Atlas database (TCGA, http://cancergenome.nih.gov/).

### Statistical analysis

The normal distribution of data was tested by the 1-sample Kolmogorov–Smirnov test. The continuous variables were expressed as mean ± SE or median (25th–75th quartile). The concentrations of plasma microRNA expression levels measured by qRT-PCR were normalized by log_2_ transformed. Multivariate regression models were used to evaluate the association between plasma microRNA levels and PM_2.5_ or PM_10_ exposure levels, with adjustment for covariates including age, gender, smoking status, drinking status and BMI. All analyses were carried out using Statistical Analysis System software (version 9.0; SAS Institute, Cary, NC). Two-side *P* < 0.05 was considered statistically significant.

## SUPPLEMENTARY FIGURE AND TABLES


