# Supplementary material for: Exposure to airborne PM2.5 suppresses microRNA expression and deregulates target oncogenes that cause neoplastic transformation in NIH3T3 cells
Source: Oncotarget. 2015 Aug 21;6(30):29428–39. doi: 10.18632/oncotarget.5005 (PMC4745737; doi:10.18632/oncotarget.5005)
Supplement: Supplementary file 1 [file oncotarget-06-29428-s001.pdf]

SUPPLEMENTARY FIGURE AND TABLES

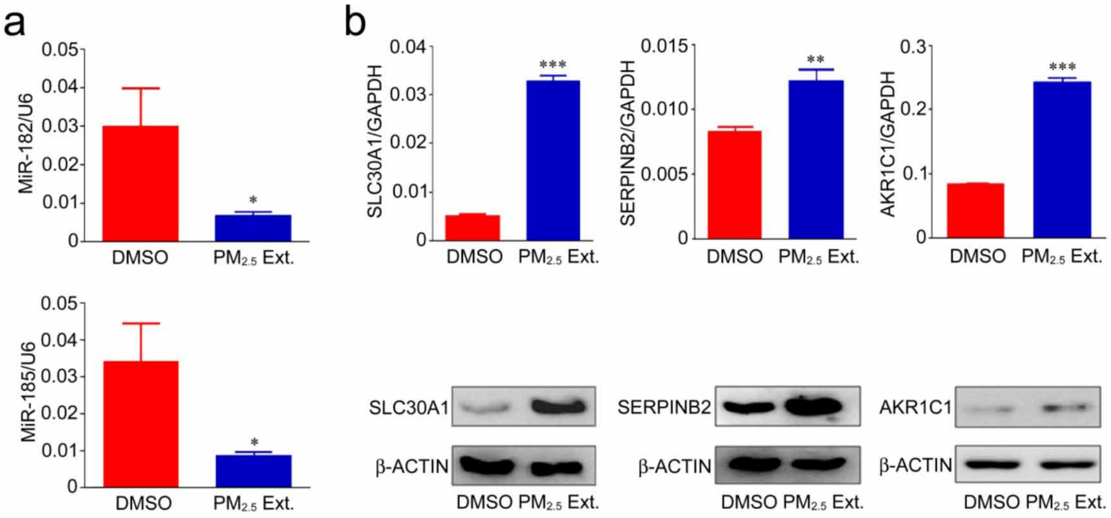

Supplementary Figure S1: Quantitative real-time PCR and Western blot analysis of differential expression of miRNA-182 and miRNA-185. a. and potential target genes *SLC30A1*, *SERPINB2* and *AKR1C1* **b.** in human bronchial epithelial cells exposed to $PM_{2.5}$ extracts (indicated as $PM_{2.5}$ Ext.). Except for Western blot, results are mean ± SEM obtained from three experiments and each had six replicates. *, $P < 0.05$, **, $P < 0.01$ and ***, $P < 0.001$ compared with DMSO control.

Supplementary Table S1: Baseline characteristics of study subjects

	$PM_{2.5}$		PM_{10}	
	**Low exposure**	**High exposure**	**Low exposure**	**High exposure**
Age (year, Mean ± SD)	51.78 ± 5.67	51.05 ± 6.79	51.50 ± 5.65	51.42 ± 6.78
**Gender, n (%)**				
Male	27 (43.5)	23 (48.9)	21 (35.0)	29 (59.2)
Female	35 (56.5)	24 (51.1)	39 (65.0)	20 (40.8)
BMI (kg/m^2, Mean ± SD)	24.37 ± 3.35	24.78 ± 3.32	24.33 ± 3.10	24.82 ± 3.62
**Smoking status, n (%)**				
Never	14 (22.6)	22 (46.8)	46 (76.7)	21 (42.9)
Ever	48 (77.4)	25 (53.2)	14 (23.3)	28 (57.1)
**Drinking status, n (%)**				
Never	14 (22.6)	17 (36.2)	45 (75.0)	22 (44.9)
Ever	48 (78.4)	30 (53.8)	15 (25.0)	27 (55.1)
**$PM_{2.5}$ (μg/m^3)**				
Mean ± SD	89.4 ± 22.7	171.6 ± 33.1	99.3 ± 36.8	156.1 ± 44.7
Range	18.7–122.4	125.7–274.2	18.7–223.7	38.7–274.2
**PM_{10} (μg/m^3)**				
Mean ± SD	145.9 ± 47.9	223.4 ± 60.2	130.6 ± 30.9	238.9 ± 44.3
Range	55.6–334.0	39.8–383.3	39.8–178.2	181.3–383.3

Supplementary Table S2: Sequences of primers used for PCR or qRT-PCR and of microRNA mimics and inhibitors used in this study

Primer used for	Sequence
**cloning 3′UTR of**	
SLC30A1	5′-CGGGGTACCAACTAAGCGTGCTGCTTCT-3′
	5′-CCGCTCGAGTATTGTTGTTTGGAAGCAGG-3′
SERPINB2	5′-CGGGGTACCGTCTTGAAAAAGATGTGATATTTG-3′
	5′-CCGCTCGAGTTCCAGTAACAATTTTATTTATCC-3′
**site-mutation of 3′UTR of**	
SLC30A1	5′-CCACTGAAATTCTAAGTATCCGATGTAGTGTAATTGAAG-3′
	5′-CTTCAATTACACTACATCGGATACTTAGAATTTCAGTGG-3′
SERPINB2	5′-CAGAATTGCTATTTCAAATAGGCTATAATTTAGAGATGTTTTCTAC-3′
	5′-GTAGAAAACATCTCTAAATTATAGCCTATTTGAAATAGCAATTCTG-3′
AKR1C1-site 1	5′-GCCTCTGGTTAAATCACACGTGCTTGGTGATTTC-3′
	5′-GAAATCACCAAGCACGTGTGATTTAACCAGAGGC-3′
AKR1C1-site 2	5′-CATTTTGAAAAAATTAAATGCTCACACGTAAAGATTCTTCACCTAAAA-3′
	5′-TTTAGGTGAAGAATCTTTACGTGTGAGCATTTAATTTTTTCAAAATG-3′
**cloning full length cDNA of**	
SLC30A1	5′-CCGCTCGAGATGGAGGATCTTTGTGTGGCAA-3′
	5′-CAGCGGCCGCTTAGGGTGAGGAAAATCTGCCG-3′
SERPINB2	5′-CCGCTCGAGATGGGGTGTTGGGGTC-3′
	5′-CAGCGGCCGCTCACAAAGATGATTCAGGTTG-3′
AKR1C1	5′-CCGCTCGAGATGGATTCGAAATATCAGTG-3′
	5′-CAGCGGCCGCTTAATATTCATCAGAAAATGGAT-3′
**qRT-PCR of mRNA of**	
SLC30A1	Primer: 5′-CCAATACCAGCAACTCCAACG-3′
	Primer: 5′-ACTGAACCCAAGGCATCTCCA-3′
SERPINB2	Primer: 5′-GGGTAGTTATCCTGATGCGATTT-3′
	Primer: 5′-TTTGCCTTTGGTTTGAGTCTTG-3′
AKR1C1	Primer: 5′-GTAAAGCTTTAGAGGCCACC-3′
	Primer: 5′-GAGGTCAACATAATCCAATTGA-3′
GAPDH	Primer: 5′-GAAGGTGAAGGTCGGAGTCAA-3′
	Primer: 5′-GCTCCTGGAAGATGGTGATG-3′
**qRT-PCR of**	
MiR-182	Primer: 5′-TTTGGCAATGGTAGAACTCACACT-3′
	Primer: 5′-GCTGTCAACGATACGCTACCTA-3′
MiR-185	Primer: 5′-TGGAGAGAAAGGCAGTTCCTGA-3′
	Primer: 5′-GCTGTCAACGATACGCTACCTA-3′

(*Continued*)

Primer used for	Sequence
U6	Primer: 5′-CTCGCTTCGGCAGCACA-3′
	Primer: 5′-GCTGTCAACGATACGCTACCTA-3′
**MicroRNA or inhibitor**	**Sequence**
MiR-182 mimic	5′-UUUGGCAAUGGUAGAACUCACACU-3′
MiR-185 mimic	5′-UGGAGAGAAAGGCAGUUCCUGA-3′
MiR-182 inhibitor	5′-AGUGUGAGUUCUACCAUUGCCAAA-3′
MiR-185 inhibitor	5′-UCAGGAACUGCCUUUCUCUCCA-3′
MicroRNA control	5′-UUCUCCGAACGUGUCACGUTT-3′
Cel-miR-39	5′-TCACCGGGTGTAAATCAG-3′

Note: 3′-UTR, 3′-untranslated region; qRT-PCR, quantitative real-time PCR.
